# Supplementary figures and images for: Variant calling and genotyping accuracy of ddRAD-seq: Comparison with 20X WGS in layers
Source: PLoS One. 2024 Jul 26;19(7):e0298565. doi: 10.1371/journal.pone.0298565 (PMC11280156; doi:10.1371/journal.pone.0298565)

### Macro-chromosomes

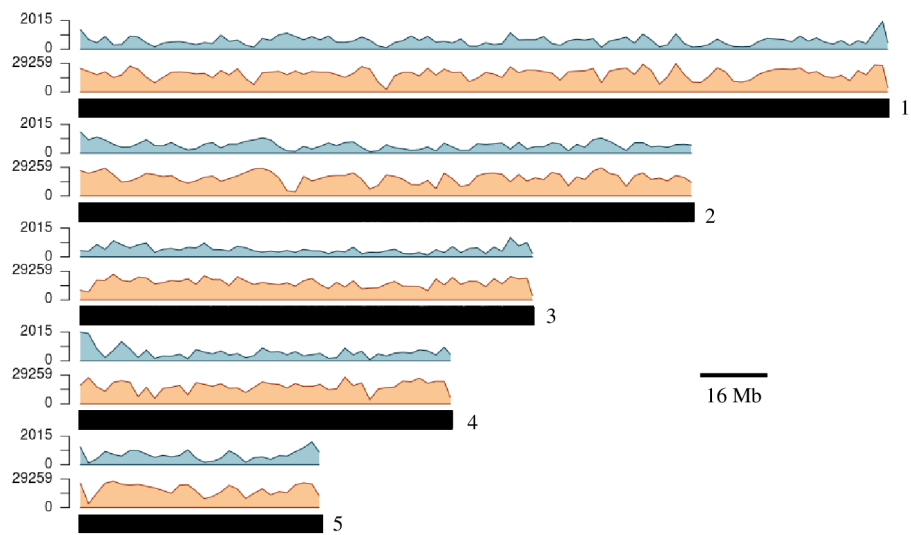

### Intermediate chromosomes

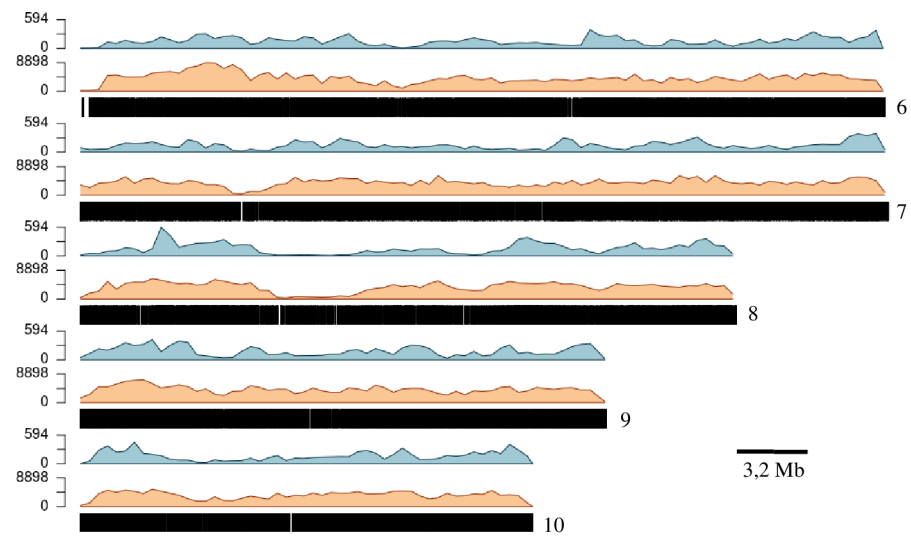

### Micro chromosomes

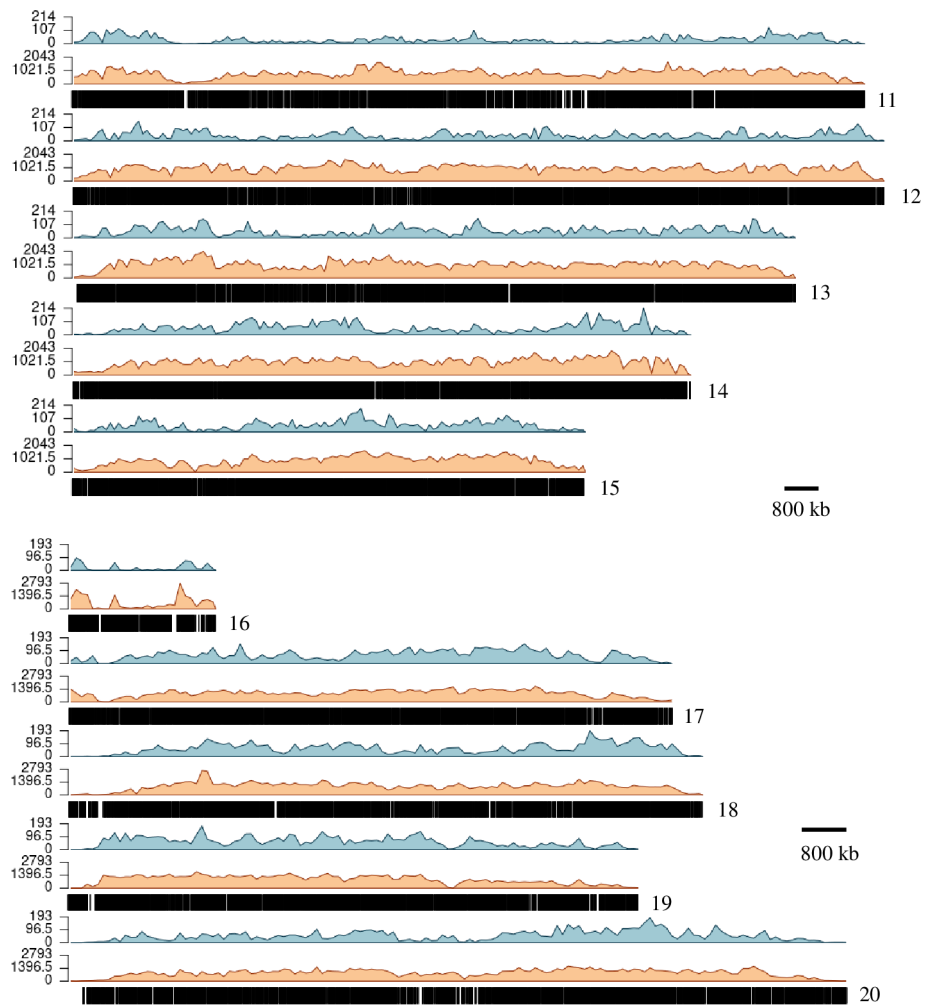

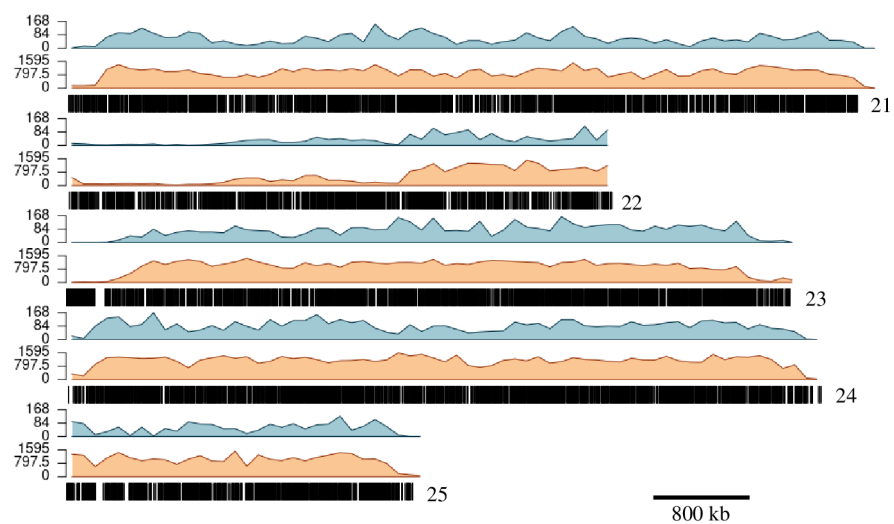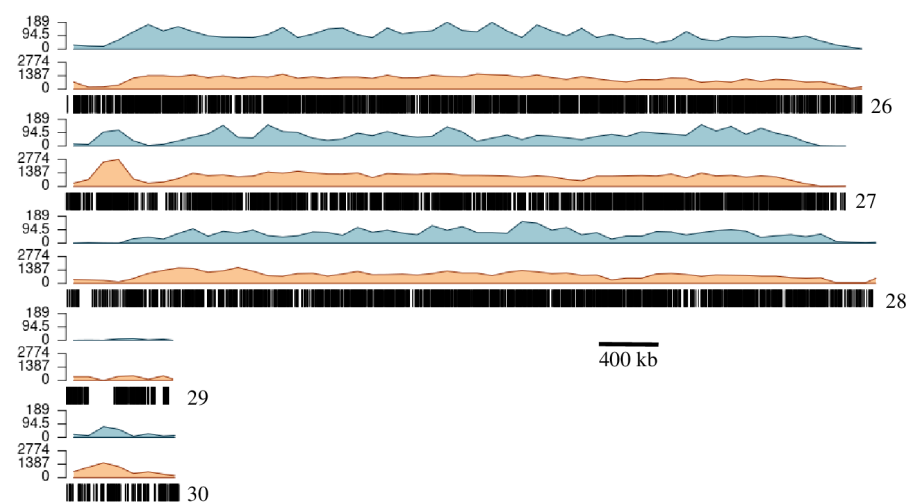

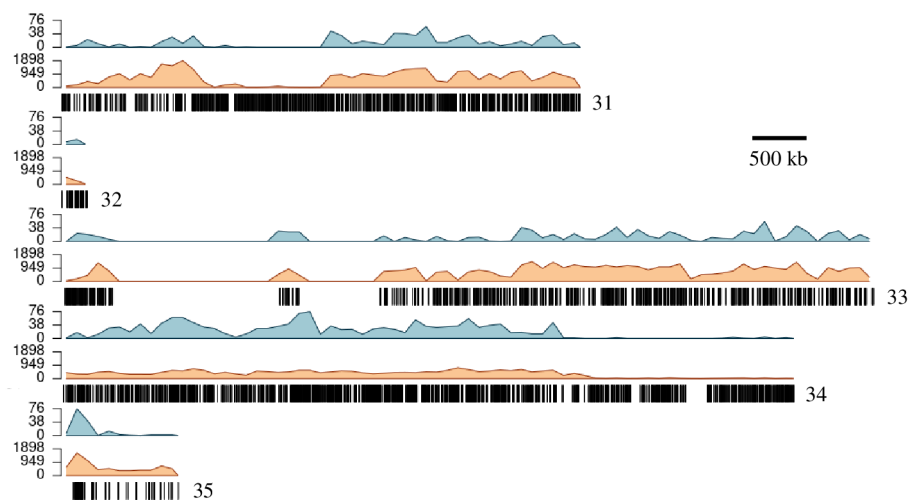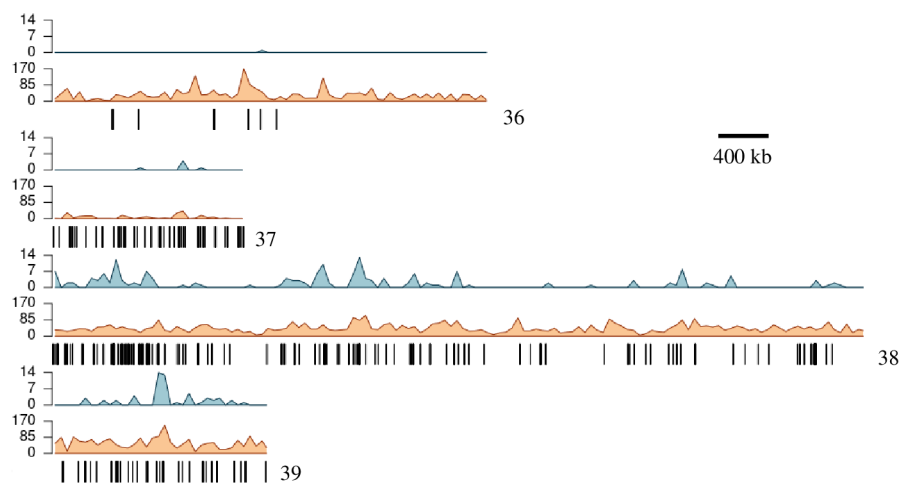

Supplement: S1 Fig — For each type of chromosome category of the chicken genome (macro-chromosome, intermediate chromosome, and micro-chromosome), the SNP distribution of the ddRAD-Seq data (in blue) and 20X experiment (in orange) is displayed. The black bar represents the theorical restriction fragment location. (PDF) [file pone.0298565.s001.pdf]

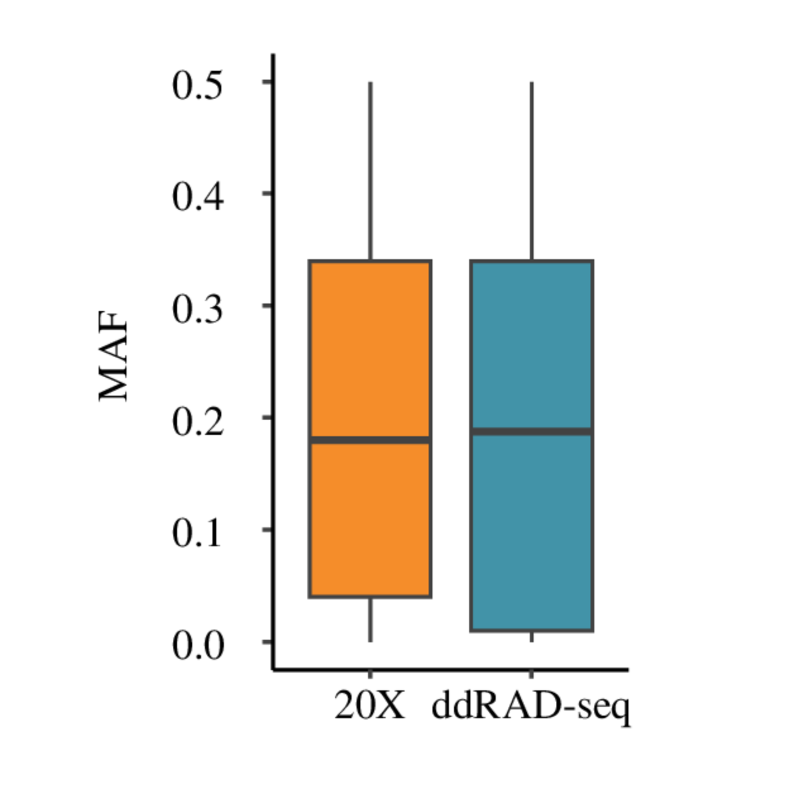

Supplement: S2 Fig — (TIF) [file pone.0298565.s002.tif]
